# Biosynthesis and Characterizations of Silver Nanoparticles from *Annona squamosa* Leaf and Fruit Extracts for Size-Dependent Biomedical Applications

**DOI:** 10.3390/nano12040616

**Published:** 2022-02-11

**Authors:** Maria Malik, Muhammad Aamir Iqbal, Mariam Malik, Muhammad Akram Raza, Wajeehah Shahid, Jeong Ryeol Choi, Phuong V. Pham

**Affiliations:** 1 Centre of Excellence in Solid State Physics, University of the Punjab, Lahore 54590, Pakistan; mariamalikc@gmail.com (M.M.); akramraza-cssp@pu.edu.pk (M.A.R.); 2 School of Materials Science and Engineering, Zhejiang University, Hangzhou 310027, China; 3 Department of Botany, Government College Women University, Sialkot 51310, Pakistan; mariyammalik2001@gmail.com; 4 Department of Physics, The University of Lahore, Lahore 54000, Pakistan; wajeeha.shahid@phys.uol.edu.pk; 5 Department of Nanoengineering, Kyonggi University, Suwon 16227, Korea; 6 Hangzhou Global Scientific and Technological Innovation Center, School of Micro-Nano Electronics, Zhejiang University, Hangzhou 310027, China

**Keywords:** green synthesis, AgNPs, *Annona squamosa*, bandgap, antibacterial, size-dependent applications

## Abstract

Green synthesis differs in the way that the plant produces chemicals that act as reducing and stabilizing agents, and by adopting this green synthesis, we have synthesized silver nanoparticles (AgNPs) from the leaf and fruit extracts of *Annona squamosa* (also known as Sharifa), where these extracts have played an important role as reducing and capping agents. The nanoparticles were synthesized as the consequence of a reduction that happened between plant extracts and the precursor solution. The prepared AgNPs were then characterized using scanning electron microscopy, UV-Visible spectroscopy, and X-ray diffraction to study their morphology, optical response, and crystallinity. A single distinctive absorption peak of colloidal AgNPs samples was observed at 430 nm and 410 nm for leaf and fruit extract samples, having an optical bandgap of 2.97 eV and 2.88 eV, respectively, with a spherical shape having a diameter in the range of 35–90 nm and 15–50 nm, respectively, whilst XRD studies supported the FCC cubic structure of the mediated AgNPs. These green synthesized AgNPs have a wide variety of uses, particularly in the biomedical domain, where they have the potential to treat numerous diseases and are reported to be efficient against antibacterial, anti-cancer, and anti-diabetic activities.

## 1. Introduction

The most significant feature of nanotechnology is to develop more reliable processes for the synthesis of nanomaterials with a wide variety of sizes, good mono-dispersity, and chemical compositions [1]. Nanomaterials of various shapes and sizes ranging from 1 nm to 100 nm are being synthesized on a large scale in laboratories because of their wide-ranging and practical uses in a variety of industries. In addition to various chemical methods, two approaches, top-down and bottom-up, are widely employed for nanoparticle synthesis [2]. However, the usage of lethal and toxic chemicals was their stumbling block [3]. During the last decade, nanotechnologists have proposed a very profitable and efficient method to synthesize nanoparticles (NPs), which is also known for its user-friendly and non-toxic nature, as well as the fact that there are no complex instruments involved in employing the green synthesis method, which also minimizes ecotoxicity [4]. The biosynthesized metallic NPs have a broad spectrum of applications in science and technology, including medicine, biotechnology, physics, chemistry, and material sciences.

In contrast to other methods that require the usage of costly and harmful chemicals as reducing, capping, and some stabilizing agents for the synthesis of NPs, green synthesis utilizes the plant itself to produce such chemicals that work as natural reducing and stabilizing agents [5]. Magnetic NPs have been reported to be biosynthesized using various plants [6,7], with plant parts, such as leaves [8], flowers [9], stem bark [10], and seeds [11] being used as nanoparticles factories. Silver nanoparticles (AgNPs) mediated by *Moringa oleifera* leaves [12], ZnO NPs mediated by *Capsicum annuum* fruit extract [13], platinum NPs mediated by date extracts [14], and many more have been reported so far [15,16,17]. Biological waste and byproducts, such as peels [16] and *Annona squamosa* leaves [17], have also been reported to be employed in the green synthesis of AgNPs, anticipating the potential of AgNPs for a number of applications and prompting researchers to further examine AgNPs synthesized by plants to investigate their potential in healthcare.

The most essential and distinctive aspect of NPs is their increased surface-area-to-volume ratio, which explains the reason behind their potential for size-dependent applications. The most efficiently explored NPs, recently, are those formed of noble metals, namely Ag, Pt, Au, and Pd, whereas AgNPs, among the four stated above, play an essential role in biology and medicine. The synthesis of AgNPs by reducing aqueous silver nitrate (AgNO_3_) with aqueous *Annona squamosa* leaf and fruit extracts provide innovative, non-toxic, environmentally friendly, as well as cost-effective reducing agents for bio-reduction of AgNO_3_, and are used in folklore medicine for the treatment of various ailments. The phytochemicals, mainly flavones, terpenoids, sugars, ketones, aldehydes, carboxylic acids, and amides, are the primary ingredients found in plants that are involved in the bio-reduction of nanoparticles [18]. Flavonoids have a variety of functional groups that boost their capacity to reduce metal ions because of the tautomeric reactions in flavonoids that transform the enol-form into the keto-form and release the reactive hydrogen atom, resulting in the conversion of metal ions into stable atoms, which then nucleate under continual heating and stirring to synthesize nanoparticles [19]. Sugars found in plant extracts, such as glucose and fructose, may also have a role in the creation of metallic nanoparticles. Glucose has the ability to play a role in the size and shape of distributed nanoparticles, whereas fructose is responsible for monodispersed silver nanoparticles [20]. The protein present in plant extracts with a functional amino group (NH_2_) also participates in reducing metal ions to stable metallic atoms [21]. Functional groups found in phytochemicals, such as flavones, alkaloids, phenols, and anthracenes (such as –C–O–C–, –C–O–, and –C=C–) can play an important role in the formation of metallic nanoparticles via metallic atom agglomeration followed by metallic ion reduction. The natural chemical compounds are present in significant quantities in *Annona squamosa*, such as flavonoids, alkaloids, potassium, ascorbic acid, amino acids, riboflavin, and acetogenins. The leaf study revealed the presence of caryophyllene, cadinol, muurolene, and isoquinoline alkaloids. Annoreticuin and isoannoreticuin are acetogenins discovered in leaves that are cytotoxic to human tumor cells [22], whereas samoquasine, benzylisoquinoline, and tertahydroisoquinoline are alkaloids identified in plant fruit extract. Further, the root and bark include liriodenine and oxoanalobine, and the bark extract has a single acetogenin, solamin, distinct bullatacinone, and two triterpenoids, making it appropriate for antisecticidal applications [23]. Quercetin, a biomolecule comprised of three rings and five OH groups, is the major biomolecule found in *Annona squamosa* extracts with the formula C_15_H_10_O_7_ [24]. Owing to the presence of these functional groups, *Annona squamosa* showed peculiarly different antimicrobial and cytotoxic accomplishments. The AgNPs derived from *Annona squamosa* plant leaf extract are of high therapeutic interest due to their efficacy against a variety of bacterial infections and are chosen over other metal NPs due to their extremely small size, improved physio-chemical characteristics, nontoxicity to human cells, and inexpensive production cost [25]. They are also well-known for their excellent antibacterial activity, despite their small size [26].

This innovative study is aimed at exploring the synthesis of AgNPs using a green synthesis method from the leaf and fruit extracts of *Annona squamosa* in order to identify their potential for biological and biomedical applications. As per our understanding, it is the first-ever study that explores *Annona squamosa* fruit extracts’ potential for being used as antibacterial treatments and provides a comparison of leaf and fruit extracts for size-dependent biomedical applications. This work revealed efficient antibacterial effects without the use of any chemical entities by maintaining the purity of the green-produced AgNPs, which is a significant advantage over prior studies [16,17]. For the first time, the antibacterial activity of various concentrations of synthesized AgNPs (25 µg/mL, 50 µg/mL, and 100 µg/mL) was investigated, as well as a comparison of their extract activities. The mechanism of bacterial cell death has also been demonstrated in detail.

## 2. Materials and Methods

Materials: Fresh *Annona squamosa* (sweet apple) fruit and leaves were obtained from the Botanical Garden (University of the Punjab, Lahore, Pakistan), and were then washed, dried (in the case of pulp fruit), and crushed into a fine powder (in the case of leaves).

Chemicals and Equipment: The only chemical, silver nitrate solution (Sigma Aldrich Inc., Lahore, Pakistan) having 99.95% purity, was used. The deionized water used was synthesized in the lab at the Centre of Excellence in Solid State Physics, at the University of the Punjab, Lahore, Pakistan. A hot plate, beakers, an electronic weight balance, a vernier caliper, Petri dishes, and magnetic bits were among the instruments used, with all the experiments taking place at room temperature.

Methods: The synthesis of AgNPs was performed by following the methods outlined below:

- Leaf and fruit extract preparation;
- Precursor solution preparation;
- Nanoparticle synthesis.

**Figure 1:**
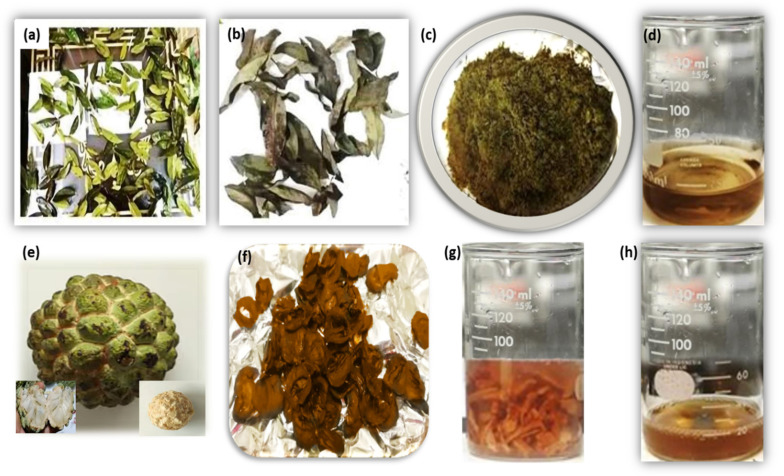
Steps involving the preparation of *Annona squamosa* from leaf extract (**a**–**d**), and fruit extract (**e**–**h**). Insets in (**e**) are the core of the fruit (left bottom) and the fruit detached from the shell (right bottom).

Leaf Extract Preparation: The collected *Annona squamosa* leaves were washed 2–3 times with fresh water to eliminate any discrepancies in results, then thoroughly dried before being crushed into powder form, and 5 g of this powder was weighed, which was then mixed with 100 mL of deionized water in a beaker containing 5 g of leaves powder. The beaker was placed on the hot plate and the solution was boiled and stirred for 25–30 min at boiling temperature before being filtered by Whatman filter paper no.1. Figure 1a–d depicts the step-by-step synthesis of the leaf extract.

Fruit Extract Preparation: The *Annona squamosa* fruit was peeled, cut, rinsed 2–3 times with fresh water to avoid any discrepancy, and then soaked for 24 h in 100 mL of deionized water. After 24 h, the beaker was placed on a hot plate, and the solution was boiled and stirred for 35–40 min at a boiling temperature before being filtered by Whatman filter paper no.1. Figure 1e–h depicts the step-by-step preparation of the fruit (pulp) extract.

**Figure 2:**
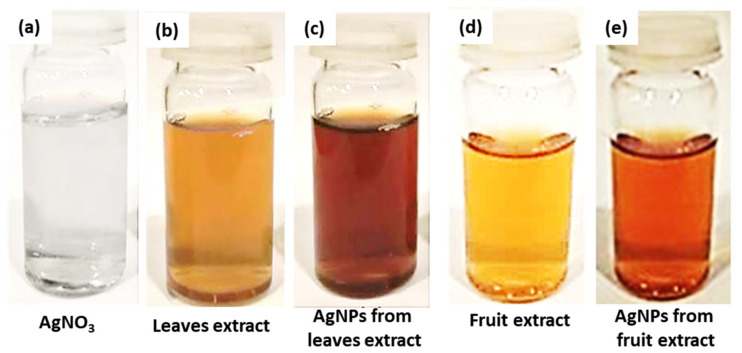
Schematic of silver nanoparticles preparation (**a**) AgNO_3_ solution, (**b**) *Annona squamosa* leaf extract, (**c**) AgNPs from leaf extract, (**d**) *Annona squamosa* fruit extract, and (**e**) AgNPs from fruit extract.

Precursor Solution Preparation: A 100 mL solution of 10 mM AgNO_3_ was prepared by dissolving 0.169 g of AgNO_3_ in 100 mL of deionized water and was employed as a precursor for both the leaf and fruits’ extract of *Annona squamosa* mediated AgNPs. The prepared solution is shown in Figure 2a.

*Annona squamosa* leaf mediated AgNPs: To make *Annona squamosa* leaf extract-mediated AgNPs, 45 mL of AgNO_3_ precursor solution was heated in a beaker, and 5 mL of *Annona squamosa* leaves extract was added dropwise into this boiling solution, which was then boiled for a further 25–30 min. Because of a surface plasmon resonance effect [27], the color changed from light yellow to light brown and then dark brown, indicating that a pH change happened, hinting at the formation of dark brown colored nano-sized AgNPs, as shown in Figure 2c, which were then stored at 4 °C for further characterizations. 

*Annona squamosa* fruit-mediated AgNPs: For the synthesis of *Annona squamosa* fruit extract-mediated AgNPs, 45 mL of AgNO_3_ precursor solution in a beaker was boiled, and 5 mL of *Annona squamosa* fruit extract was added dropwise into this boiling solution, which was then boiled further for 30–35 min at 80 °C, after which the color changed from transparent to light yellow and then to light brown. This action was caused by the surface plasmon resonance effect [27], which suggested that a pH change occurred, indicating the creation of light brown colored nano-sized silver particles, as seen in Figure 2e, which were preserved at 4 °C for subsequent characterizations.

Figure 2a–e depicts the final solution colors in the stepwise synthesis of NPs, and these synthesized AgNPs from leaf and fruit extracts were then analyzed using characterization techniques, such as scanning electron microscopy (SEM, S3400N, Hitachi Analysis Machine, Tokyo, Japan), X-ray diffraction (Diffractometer: Bruker D8 Advance Diffractometer, Bremen, Germany), and UV-Vis spectroscopy (Shimadzu UV-1800, Shimadzu Corp., Kyoto, Japan).

Protocols for Antibacterial Activity: The antibacterial potency of *Annona squamosa* leaf and fruit extracts, along with their mediated AgNPs, were analyzed against Gram-negative bacteria; *Escherichia coli* and *Pseudomonas aeruginosa* were provided by the Institute of Molecular Biology and Biotechnology, University of Lahore, Lahore Pakistan. The Muller Hinton Agar was prepared and poured in Petri dishes until it gains a jelly-like texture, then the bacterial strains were sub-cultured in this solution by the disk diffusion method to evaluate antibacterial activity. The five wells of 10 mm diameter each were created to pore solutions in them, such as deionized water, *Annona squamosa* leaf, as well as fruit extracts and AgNPs synthesized from both extracts with varying concentrations (25 µg/mL, 50 µg/mL, and 100 µg/mL) in each well 1 to 5, respectively. These dishes were then incubated at 37 °C for 24 h to analyze the zone of inhibition (ZOI) of each extract and AgNPs, with the help of a vernier caliper to measure the bacterial growth inside the respective well.

## 3. Results and Discussions

### 3.1. Synthesis of Nanoparticles (NPs)

This study focused on the synthesis of AgNPs, which are more advantageous owing to their small size and nontoxicity to human cells but toxic to microorganisms, such as bacteria [28]. Because of these characteristics, AgNPs have special physical, chemical, and optical properties that are important in bio-nanotechnology [29]. Green synthesis provides another source for the synthesis of NPs, such as AgNPs [30], in which the plant and fruit extract itself are employed as a reducing agent, converting metal ions into stable metal NPs [31].

**Figure 3:**
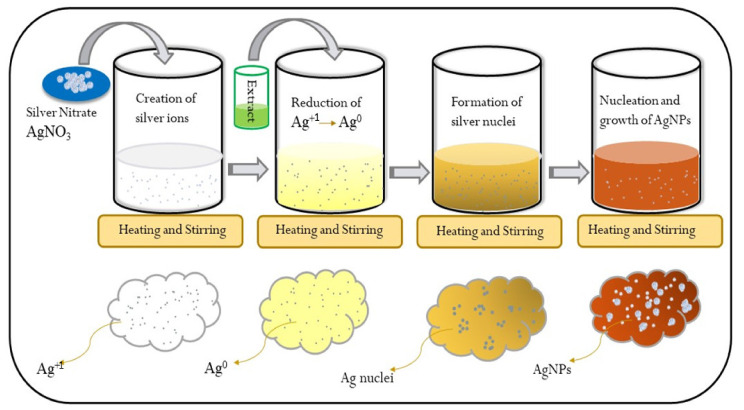
A synthesis mechanism of extract mediated AgNPs.

A reduction process occurred when fruit and leaf extracts were added to the precursor solution, resulting in the creation of AgNPs [32]. Figure 3 depicts a schematic mechanism of AgNPs synthesis in which AgNO_3_ was dissolved in deionized water with no color, boiled, and stirred on a hot plate at a boiling temperature prior to the addition of *Annona squamosa* leaf/fruit extract into the beaker. After the addition of the extract solution, it acted as a reducing and stabilizing agent, converting Ag^+1^ ions to Ag^0^ particles on continuous heating and stirring, which is the basis of silver nuclei formation, and could also be visualized by the solution color changing. Following the start of the nucleation process, additional heating and stirring contributed to the nucleation and growth processes, which resulted in the synthesis of the required AgNPs, as indicated by the golden brown and light brown colors of the final solution treated with leaf and fruit extracts, respectively. Herein, phenol and other reducing agents found in citrus extracts were the primary sources of reducing Ag^+1^ ions to Ag^0^ particles, and hence the observed color transformation was attributed to a reduction mechanism [33]. By manipulating the concentrations of extracts and precursor solutions, the ultimate size of the produced AgNPs may be adjusted [34]. Recent research has shown the shapes, size ranges, and structures of AgNPs as described by several methods [35].

### 3.2. Characterizations

#### 3.2.1. X-ray Diffraction (XRD) Analysis

The structural analysis of synthesized AgNPs was performed by XRD to investigate a material’s purity, crystal structure, crystal phase identification, lattice parameter, crystallite size, and crystal defects [36,37]. It is a nondestructive technique that is based on the coherent scattering mechanism, where the sample is collimated and electrons penetrate the inner transition, producing X-rays that are then thrown over the sample, rotated at different angles, and hence the intensity of reflected X-rays is recorded at these different angles. When the X-rays satisfy Bragg’s equation, an intensity peak is obtained [38], which is further investigated to explore structural parameters. The XRD pattern of AgNPs was recorded by an X-ray Powder Diffractometer (Bruker D8 Advance Diffractometer, Bremen, Germany).

**Figure 4:**
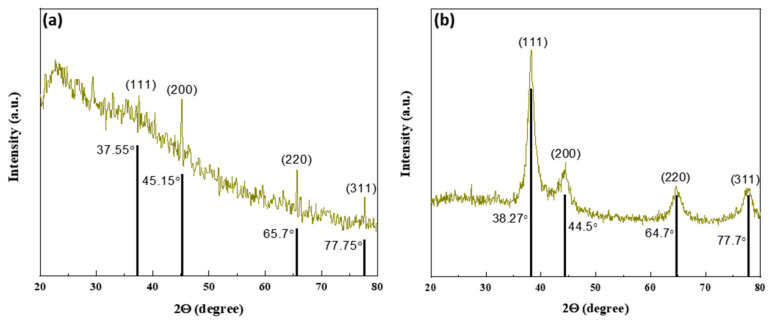
XRD patterns of *Annona squamosa* mediated AgNPs along with standard silver patterns (**a**) leaf extract, and (**b**) fruit extract.

The XRD data were collected by a Bruker D8 advanced with Cu-kα radiation as an X-ray source, having a wavelength of 1.5406 Å. The pattern was obtained in the range of 20° and 80° of 2θ values with a step width of 2θ = 0.04, as shown in Figure 4. As we can see, peaks appear in the pattern at different 2θ values, which confirmed the presence of AgNPs demonstrating the hkl values of (111), (200), (220), and (311) at 2θ values of 37.55°, 45.15°, 65.7° and 77.75°, respectively, in the case of leaf mediated AgNO_3_, as shown in Figure 4a, while the same planes were observed in fruit-mediated AgNPs, as shown in Figure 4b, with a slight change in 2θ values as 38.27°, 44.5°, 64.7° and 77.7°, respectively. These results confirm that the green synthesized AgNPs are face-centered cubical, indicating that they are crystalline in nature [39], and when these patterns were compared to JCPDS ID No. 04-0783, it was discovered that they were silver, and more specifically, AgNPs.

Stress and strain have an effect on the crystal lattice, and instrumental modifications can influence peak width, whereas full-width half maxima (FWHM), which is denoted by β was calculated using the major peak of the 111 plane in the XRD pattern and calculated to be 0.0069 and 0.0197 for leaf and fruit extracts mediated AgNPs, respectively. The Bragg’s law [40] was used to calculate the lattice parameter, while the Debye–Scherrer equation [41] was used to measure the crystallite size of the synthesized AgNPs as given below.

(1) $$D=\frac{\delta \lambda }{\beta \;Cos\theta }$$

Table 1 shows the measured structural parameters, with the calculated crystallite size of *Annona squamosa* mediated AgNPs being 21.4 nm for leaf extract and 8.0 nm for fruit extract, respectively, indicating a good affinity with our estimated UV-Vis results.

#### 3.2.2. UV-Vis Spectroscopy Analysis

UV-Vis spectroscopy is a frequent and successful approach for studying the optical responses of colloidal NPs due to the narrow bandgap between the valance and conduction bands of metallic NPs such as AgNPs, where electrons can freely move between them. Surface plasmon resonance (SPR) is a resonant oscillation of conduction electrons caused by electrons on the surface of AgNPs in response to incoming light. Colloidal NPs produce a variety of colors as a result of SPR absorption, depending on various factors, such as size, shape, and the surrounding medium [38]. A Shimadzu UV-1800 (Japan) UV-Vis spectroscopy model was employed, which provided us with graphical data shown in relation to wavelength (along the *x*-axis) and absorbance (along the *y*-axis). This analysis was used to confirm whether or not we have synthesized the required particles by providing information about the absorption peaks [32]. AgNPs differ from other NPs in that they exhibit a surface plasmon resonance effect [42], which occurs when light interacts with conduction electrons present on the exposed metal surface, resulting in collective electronic oscillations in response to specific light wavelengths [43].

**Figure 5:**
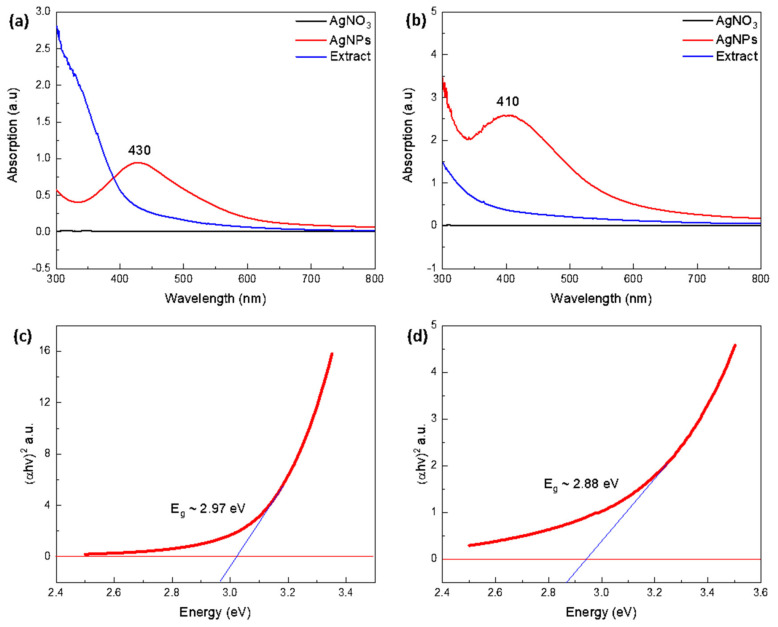
UV-Vis absorption spectrum for mediated AgNPs of (**a**) leaf extract, (**b**) fruit extract, and optical bandgap of (**c**) leaf extract, and (**d**) fruit extract. Note: Blue lines in (**c**,**d**) indicate the slopes which are used to find the optical bandgap (*E_g_*) values (2.97 and 2.88 eV), respectively.

As shown in Figure 5a,b, the UV-Vis analysis displays a graph of leaf and fruit-mediated AgNPs, with the wavelength along the *x*-axis and absorbance along the *y*-axis, where the AgNO_3_ precursor solution and leaf/fruit extract did not show any absorption peaks; however, a peak at 430 nm and 410 nm indicated the presence of AgNPs in the sample solution due to the surface plasmon resonance effects. According to the literature, AgNPs often have UV-Vis peaks in the 400–500 nm wavelength range, depending on their size [44]. Furthermore, the presence of a single absorption peak suggested the development of spherical-shaped AgNPs, which were investigated further using morphological SEM analysis [45]. Moreover, the optical bandgap of leaf and fruit-mediated AgNPs was computed by Tauc’s plot [46] using the relation:

(2) $$\alpha h\vartheta =A\;{\left(h\vartheta -E_{g}\right)}^{n}$$

where *α* represents the absorption coefficient, *hν* is the photon energy, *A* is a constant, and *E_g_* is the optical bandgap energy, respectively, while *n* depends on electron transitions, and *n* = 2 corresponds to direct electron transitions with a direct optical bandgap. The graph between (*αhν*)^2^ and *E_g_*, as shown in Figure 5c,d, estimates the optical bandgap energy of synthesized AgNPs as 2.97 eV and 2.88 eV using leaf (Figure 5c) and fruit extracts (Figure 5d), respectively, which are comparable with existing literature at 2.9 eV [47], and 3.4 eV [48]. These results are more significant, and minor variations in the optical bandgap may be attributable to quantum confinement effects [49].

#### 3.2.3. Scanning Electron Microscope (SEM) Analysis

**Figure 6:**
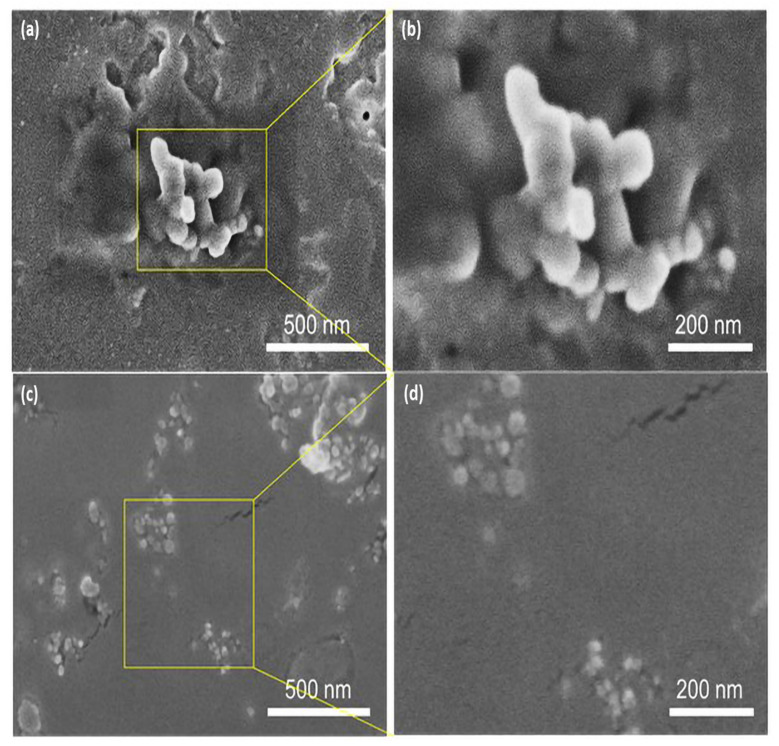
SEM analysis mediated AgNPs (**a**) leaf extract at a scale of 500 nm, (**b**) leaf extract at a scale of 200 nm, (**c**) fruit extract at a scale of 500 nm, and (**d**) fruit extract at a scale of 200 nm.

SEM is a popular and widely used technique for evaluating the shape, size distribution, surface morphology, and elemental composition of nanomaterials. The AgNPs samples were prepared for SEM analysis by cleaning the Si wafer slides with IPA and ethanol first, then placing drops of the sample on the slides and allowing them to dry. The samples were then mounted on double carbon tape to provide a conductive medium for SEM imaging, while the crystallite size was measured using the Debye–Scherrer formula. SEM analysis was utilized to explore the shape of the leaf and fruit extract-mediated AgNPs by evaluating micrographs at 500 nm and 200 nm scales, as shown in Figure 6a–d. The leaf extract and fruit extract-mediated AgNPs yielded micrographs that display the particle size distribution as well as the shape [50], and offer a histogram by physically counting the particles or using specialist software [51]. The analysis of *Annona squamosa* leaf extract-mediated AgNPs revealed that the particle size of silver was in the range of 35–90 nm, whereas fruit extract-mediated AgNPs exhibited a range of 15–50 nm and demonstrated their spherical shape, which generally depends on various factors, such as pH level, temperature, reaction time, extract, and precursor solution concentrations, which, as a whole, identifies the reason for the different sizes of AgNPs [52]. The size distribution of silver nanoparticles can be minimized by adjusting the operational parameters prior to syntheses, such as temperature, reaction time, precursor concentration, and extract quantity.

**Figure 7:**
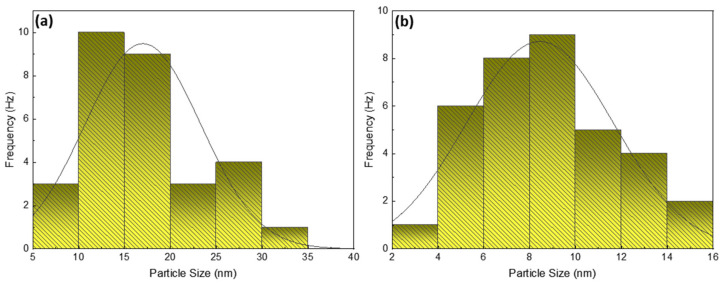
Histogram depicting size and frequency of mediated AgNPs particles at a scale of 200 nm (**a**) leaf extract (**b**) fruit extract.

Small spherical-shaped silver particles, like bright tiny spots, can be seen on the extract’s larger bio-particles in the case of AgNPs generated by leaf extract, while the agglomeration of a few smaller particles into small clusters can also be seen in Figure 6a. Smaller NPs have high surface energy and agglomerate into larger-sized particles to lower their surface energy [53]. The size distribution range of these spherical AgNPs was determined to be 35–90 nm, as seen in Figure 6b’s magnified view of the 200 nm scale and graphically displayed in Figure 7a of particle size histogram. The fruit extract-mediated AgNPs also exhibited a spherical shape but with tiny bright spots, with a bit less agglomeration effect compared to leaf extract-mediated AgNPs (Figure 6c), whereas a magnified view with a scale of 200 nm is demonstrated in Figure 6d. The smaller size of particles confirmed the low surface energy and hence, high throughput with a size distribution of 15–50 nm, as presented in the histogram in Figure 7b.

## 4. Antibacterial Activity

The AgNPs produced from *A. indica* and *C. colocynphis* extracts have been found to be effective for larvicidal activity (anti-dengue) with size ranges of 11–21 nm and 21–31 nm, respectively [54]. As the present study explored distinct extracts, it discovered a size range of 35–90 nm and 15–50 nm, with the larger size showing the agglomeration effect caused by various factors, such as extract concentration, pH value, temperature, time, and so on, while the smaller size range shows a significant structure with less surface energy, and thus particles with a small size can be more effective for anti-cancer, antibacterial, and anti-larvicidal applications [54]. Additionally, the antibacterial activity of different leaf extract-mediated AgNPs with sizes ranging from 3–15 nm has been established [29], indicating a large potential for future prospects in biomedical applications. Moreover, *Malachra Capitata* leaf extract was also reported to synthesize spherical polydisperse AgNPs with sizes ranging from 5–70 nm, and the presence of natural functional chemicals was confirmed by FTIR analysis, demonstrating its potential for further antimicrobial properties [50]. This technology is potentially exciting for the large-scale synthesis of various inorganic materials due to the applications of such environmentally friendly NPs in antibacterial, wound healing, and other medical applications, and toxicity studies on human pathogens by AgNPs pave the way for a new class of antibacterial medicines.

### 4.1. Antibacterial Activity Results

**Figure 8:**
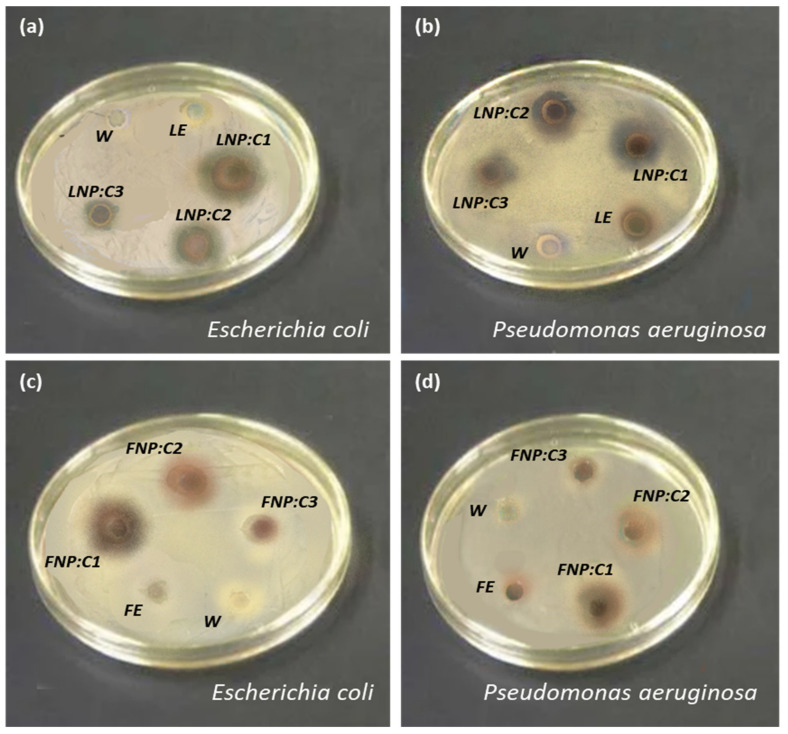
Antibacterial activity of *Annona squamosa* (**a**,**b**) leaf extract and mediated AgNPs against *E. coli*, *P. aeruginosa*, (**c**,**d**) fruit extract, and mediated AgNPs against *E. coli*, *P. aeruginosa*; where, W = water; LE = leaf extract; FE = fruit extract; LNP = leaf extract-mediated AgNPs; FNP = fruit extract-mediated AgNPs. C = concentration; C1 = 100 µg/mL, C2 = 50 µg/mL, C3 = 25 µg/mL, respectively.

The antibacterial evaluation of employed *Annona squamosa* leaf and fruit extracts along with their mediated AgNPs is described in Table 2 and illustrated in Figure 8. The results revealed that plant extracts and their mediated AgNPs were both efficacious against bacterial growth, with high concentrations of AgNPs being more appealing to both bacterial strains than their counterparts.

### 4.2. Antibacterial Mechanism

**Figure 9:**
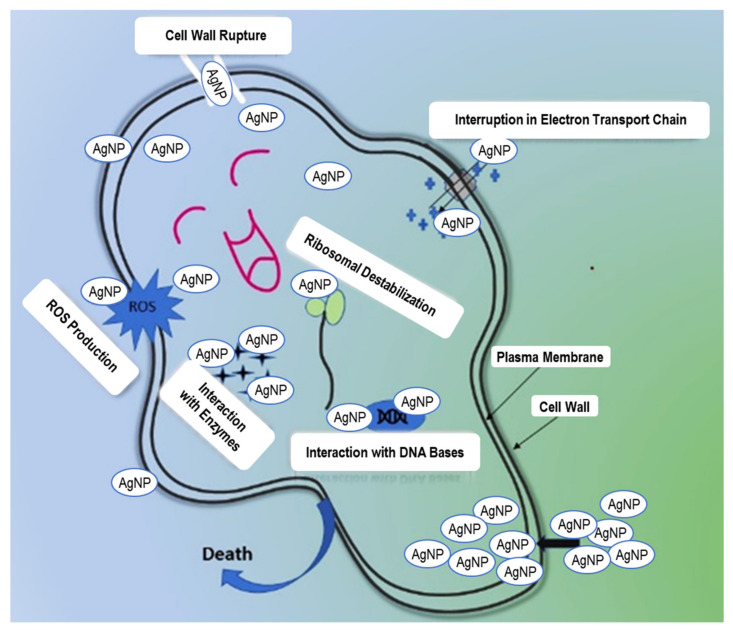
Possible antibacterial mechanisms for AgNPs.

Silver is a widely used antibacterial agent that can kill over 650 distinct microorganisms, including gram-negative and gram-positive bacteria, fungi, and viruses, demonstrating medicinal effects for about 2000 years [55]. Silver is commonly employed in the nitrate form to generate antimicrobial effects; however, silver nanoparticles have a significant exposed surface area for microorganisms’ interaction. Silver must be in ionized form to have an antibacterial capability where it shows inert properties, and upon interacting with moisture/target, it releases silver ions [56]. AgNPs can attack bacteria in various ways, such as the electrostatic interaction between Ag^+1^ and negatively charged bacterial cell plasma membranes, causing cell death [57]. This interaction is due to oxidative stress produced by reactive oxygen species (ROS) in the bacterial cell membrane [58]. Furthermore, when AgNPs impregnate the bacterial cell membrane by disrupting the respiratory chains, they then seep into the organelles and react with DNA, ribosomes, and enzymes where the released Ag^+1^ ions can form complex compounds with nucleic acids and primarily interact with nucleosides rather than phosphate groups, causing denaturation of the genetic material and apoptosis after binding with their components. The AgNPs also accumulate in the bacterial membranes of E. coli and P. aeruginosa cells, causing enhanced permeability and cell death. This schematic mechanism is illustrated in Figure 9.

## 5. Conclusions

In this study, we have summarized the impact of *Annona squamosa* as a reducing and capping agent that helps to reduce metal NPs such as silver and successfully synthesized AgNPs using leaf and fruit extracts by adopting a green synthesis method, which is an eco-friendly, cost-effective, non-hazardous, and speedy method. Flavonoids, phenolic compounds, quercetin, alkaloids, acetogenins, carotenoids, and other phytochemicals found in *Annona squamosa* extracts are responsible for their reducing behavior, as well as their biological applications. The size ranges of AgNPs mediated by leaf and fruit extracts were found to be 35–90 nm and 15–50 nm, respectively, with both displaying an FCC crystal structure. The findings demonstrate that both extracts and their mediated AgNPs were effective against bacterial growth, with high concentrations of AgNPs being more appealing to both bacterial strains than their counterparts, predicting a large potential for industrial applications, such as antibacterial rugs, wound healing, anti-cancer activity against various cell lines, textiles, paints, implantable medical devices, and many more, based on their size and shape.

## Figures and Tables

**Table 1:** Lattice parameter and crystallite size of synthesized AgNPs.

Sample	Two Theta (2θ) (Degrees)	FWHM β (Rad)	Miller INDICES	Lattice Constant (nm)	Crystallite Size (nm)
*Annona squamosa* leaf extract mediated AgNPs	37.5°	0.0069	(111)	1.34	21.4
*Annona squamosa* fruit extract mediated AgNPs	38.2°	0.0197	(111)	0.539	8.0

**Table 2:** Antibacterial activity of *Annona squamosa* leaf and fruit extract-mediated silver nanoparticles by forming ZOI.

Samples	Concentration of Nanoparticles	Zone of Inhibition (ZOI)	
		*E. coli*	*P. aeruginosa*
*Annona squamosa* leaf extract-mediated Silver Nanoparticles	100 µg/mL	19.3 mm	18.8 mm
	50 µg/mL	16.5 mm	16.1 mm
	25 µg/mL	15.7 mm	14.6 mm
*Annona squamosa* fruit extract-mediated Silver Nanoparticles	100 µg/mL	16.2 mm	16.8 mm
	50 µg/mL	15.4 mm	16.0 mm
	25 µg/mL	13.6 mm	14.8 mm

## Data Availability

Not applicable.

## References

[1] Rai M., Gade A., Yadav A. (2011). Biogenic nanoparticles: An introduction to what they are, how they are synthesized and their applications. Metal Nanoparticles in Microbiology.

[2] Christian P., Von Der Kammer F., Baalousha M., Hofmann T. (2008). Nanoparticles: Structure, properties, preparation and behaviour in environmental media. Ecotoxicology.

[3] Shabnam T., Behboodi Z. (2016). Comparing green chemical methods and chemical methods for the. Int. J. Pharm. Sci. Res..

[4] Naveed Ul Haq A., Nadhman A., Ullah I., Mustafa G., Yasinzai M., Khan I. (2017). Synthesis Approaches of Zinc Oxide Nanoparticles: The Dilemma of Ecotoxicity. J. Nanomater..

[5] Shaikh A.E., Satardekar K.V., Khan R.R., Tarte N.A., Barve S.S. (2018). Silver nanoparticles: Green synthesis using *Phoenix dactylifera* fruit extract, characterization, and anti-oxidant and anti-microbial activities. Appl. Nanosci..

[6] Li S., Shen Y., Xie A., Yu X., Qiu L., Zhang L., Zhang Q. (2007). Green synthesis of silver nanoparticles using *Capsicum annuum* L. Extract. Green Chem..

[7] Bai H., Zhang Z., Guo Y., Jia W. (2009). Biological synthesis of size-controlled cadmium sulfide nanoparticles using immobilized *Rhodobacter sphaeroides*. Nanoscale Res. Lett..

[8] Carmona E.R., Benito N., Plaza T., Recio-Sánchez G. (2017). Green synthesis of silver nanoparticles by using leaf extracts from the endemic *Buddleja globosa* hope. Green Chem. Lett. Rev..

[9] Sreekanth T.V.M., Duk Lee K. (2012). Green Synthesis of Silver Nanoparticles from *Carthamus tinctorius* Flower Extract and Evaluation of their Antimicrobial and Cytotoxic Activities. Curr. Nanosci..

[10] Burlacu E., Tanase C., Coman N.A., Berta L. (2019). A review of bark-extract-mediated green synthesis of metallic nanoparticles and their applications. Molecules.

[11] Tho N.T.M., An T.N.M., Tri M.D., Sreekanth T.V.M., Lee J.-S., Nagajyothi P.C., Lee K.D. (2013). Green synthesis of silver nanoparticles using *Nelumbo nucifera* seed extract and its antibacterial activity. Acta Chim. Slov..

[12] Sathyavathi R., Bala Murali Krishna M., Narayana Rao D. (2011). Biosynthesis of silver nanoparticles using *Moringa oleifera* leaf extract and its application to optical limiting. J. Nanosci. Nanotechnol..

[13] Lalithamba H.S., Raghavendra M., Uma K., Yatish K.V., Mousumi D., Nagendra G. (2018). *Capsicum annuum* fruit extract: A novel reducing agent for the green synthesis of ZnO nanoparticles and their multifunctional applications. Acta Chim. Slov..

[14] Al-Radadi N.S. (2019). Green synthesis of platinum nanoparticles using Saudi’s Dates extract and their usage on the cancer cell treatment. Arab. J. Chem..

[15] Rauwel P., Küünal S., Ferdov S., Rauwel E. (2015). A review on the green synthesis of silver nanoparticles and their morphologies studied via TEM. Adv. Mater. Sci. Eng..

[16] Kakakhel M.A., Saif I., Ullah N., Faisal S., Anwar Z., Zaheer Ud Din S. (2021). Waste fruit peel mediated synthesis of silver nanoparticles and its antibacterial activity. BioNanoScience.

[17] Kalyani R.L., Vijaykumar P.P.N., Pammi S.V.N., Rajkumar M., Swamy P.V., Murthy K.V.R. (2019). Biosynthesis of silver nanoparticles using *Annona squamosa* leaf extract with synergistic antibacterial activity. Indian J. Pharm. Sci..

[18] Mathew L., Chandrasekaran N., Mukherjee A. (2010). Biomimetic Synthesis of Nanoparticles: Science, Technology & Applicability.

[19] Ahmad N., Sharma S., Alam M.K., Singh V.N., Shamsi S.F., Mehta B.R., Fatma A. (2010). Rapid synthesis of silver nanoparticles using dried medicinal plant of basil. Colloids Surf. B Biointerfaces.

[20] Panigrahi S., Kundu S., Ghosh S., Nath S., Pal T. (2004). General method of synthesis for metal nanoparticles. J. Nanoparticle Res..

[21] Ishak N.A.I.M., Kamarudin S.K., Timmiati S.N. (2019). Green synthesis of metal and metal oxide nanoparticles via plant extracts: An overview. Mater. Res. Express.

[22] Pandey N., Barve D. (2011). Phytochemical and pharmacological review on *Annona squamosa* Linn. Int. J. Res. Pharm. Biomed. Sci..

[23] Yu J.G., Luo X.Z., Sun L., Li D.Y., Huang W.H., Liu C.Y. (2005). Chemical constituents from the seeds of *Annona squamosa*. Yao Xue Xue Bao Acta Pharm. Sin..

[24] Anand David A.V., Arulmoli R., Parasuraman S. (2016). Overviews of biological importance of quercetin: A bioactive flavonoid. Pharmacogn. Rev..

[25] Lee S.H., Jun B.H. (2019). Silver nanoparticles: Synthesis and application for nanomedicine. Int. J. Mol. Sci..

[26] Neelu C. (2018). Silver nanoparticles: Synthesis, characterization and applications. Silver Nanoparticles: Fabrication, Characterization and Applications.

[27] Krishnaraj C., Jagan E.G., Rajasekar S., Selvakumar P., Kalaichelvan P.T., Mohan N. (2010). Synthesis of silver nanoparticles using *Acalypha indica* leaf extracts and its antibacterial activity against water borne pathogens. Colloids Surf. B Biointerfaces.

[28] Sorescu A.-A., Nuţă A., Rodica M., Bunghez I. Green synthesis of silver nanoparticles using plant extracts. Proceedings of the 4th International Virtual Conference on Advanced Scientific Results.

[29] Okafor F., Janen A., Kukhtareva T., Edwards V., Curley M. (2013). Green synthesis of silver nanoparticles, their characterization, application and antibacterial activity. Int. J. Environ. Res. Public Health.

[30] Yasir M., Singh J., Tripathi M.K., Singh P., Shrivastava R. (2017). Green synthesis of silver nanoparticles using leaf extract of common arrowhead houseplant and its anticandidal activity. Pharmacogn. Mag..

[31] Iravani S. (2011). Green synthesis of metal nanoparticles using plants. Green Chem..

[32] Yusof K.N., Alias S.S., Harun Z., Basri H., Azhar F.H. (2018). *Parkia speciosa* as Reduction Agent in Green Synthesis Silver Nanoparticles. ChemistrySelect.

[33] Johnson I., Prabu H.J. (2015). Green synthesis and characterization of silver nanoparticles by leaf extracts of *Cycas circinalis*, *Ficus amplissima*, *Commelina benghalensis* and *Lippia nodiflora*. Int. Nano Lett..

[34] Sujitha V., Murugan K., Paulpandi M., Panneerselvam C., Suresh U., Roni M., Nicoletti M., Higuchi A., Madhiyazhagan P., Subramaniam J. (2015). Green-synthesized silver nanoparticles as a novel control tool against dengue virus (DEN-2) and its primary vector *Aedes aegypti*. Parasitol. Res..

[35] Azhar M.F.H. (2016). Mixed Matrix Polymer Membrane Incorporated with Biosynthesize Additive for Water Separation Process. Ph.D. Thesis.

[36] Bunaciu A.A., UdriŞTioiu E.G., Aboul-Enein H.Y. (2015). X-ray diffraction: Instrumentation and applications. Crit. Rev. Anal. Chem..

[37] Rajeshkumar S., Bharath L.V. (2017). Mechanism of plant-mediated synthesis of silver nanoparticles—A review on biomolecules involved, characterization and antibacterial activity. Chem. Biol. Interact..

[38] Barbara L.D., Christine M.C. X-ray Powder Diffraction (XRD). https://serc.carleton.edu/research_education/geochemsheets/techniques/XRD.

[39] Mehta B.K., Chhajlani M., Shrivastava B.D. (2017). Green synthesis of silver nanoparticles and their characterization by XRD. J. Phys..

[40] Jauncey G.E.M. (1924). The scattering of X-rays and Bragg’s law. Proc. Natl. Acad. Sci. USA.

[41] Holzwarth U., Gibson N. (2011). The Scherrer equation versus the ‘Debye-Scherrer equation’. Nat. Nanotechnol..

[42] Amendola V., Bakr O.M., Stellacci F. (2010). A study of the surface plasmon resonance of silver nanoparticles by the discrete dipole approximation method: Effect of shape, size, structure, and assembly. Plasmonics.

[43] Westcott S.L., Oldenburg S.J., Lee T.R., Halas N.J. (1998). Formation and adsorption of clusters of gold nanoparticles onto functionalized silica nanoparticle surfaces. Langmuir.

[44] Ashraf J.M., Ansari M.A., Khan H.M., Alzohairy M.A., Choi I. (2016). Green synthesis of silver nanoparticles and characterization of their inhibitory effects on AGEs formation using biophysical techniques. Sci. Rep..

[45] Raza M.A., Kanwal Z., Rauf A., Sabri A.N., Riaz S., Naseem S. (2016). Size- and shape-dependent antibacterial studies of silver nanoparticles synthesized by wet chemical routes. Nanomaterials.

[46] Feng Y., Lin S., Huang S., Shrestha S., Conibeer G. (2015). Can Tauc plot extrapolation be used for direct-band-gap semiconductor nanocrystals?. J. Appl. Phys..

[47] Thirumagal N., Jeyakumari A.P. (2020). Structural, Optical and Antibacterial Properties of Green Synthesized Silver Nanoparticles (AgNPs) Using *Justicia adhatoda* L. Leaf Extract. J. Clust. Sci..

[48] Abdalameer N.K., Khalaph K.A., Ali E.M. (2021). Ag/AgO nanoparticles: Green synthesis and investigation of their bacterial inhibition effects. Mater. Today Proc..

[49] Das A.J., Kumar R., Goutam S.P. (2016). Sunlight Irradiation Induced Synthesis of Silver Nanoparticles using Glycolipid Bio-surfactant and Exploring the Antibacterial Activity. J. Bioeng. Biomed. Sci..

[50] Srirangam G.M., Parameswara Rao K. (2017). Synthesis and charcterization of silver nanoparticles from the leaf extract of *Malachra capitata* (L.). Rasayan J. Chem..

[51] Fissan H., Ristig S., Kaminski H., Asbach C., Epple M. (2014). Comparison of different characterization methods for nanoparticle dispersions before and after aerosolization. Anal. Methods.

[52] Dada A.O., Adekola F.A., Adeyemi O.S., Bello O.M., Oluwaseun A.C., Awakan O.J., Grace F.-A.A. (2018). Exploring the Effect of Operational Factors and Characterization Imperative to the Synthesis of Silver Nanoparticles. Silver Nanoparticles: Fabrication, Characterization and Applications.

[53] Mehmood A., Murtaza G., Bhatti T.M., Kausar R. (2017). Phyto-mediated synthesis of silver nanoparticles from *Melia azedarach* L. leaf extract: Characterization and antibacterial activity. Arab. J. Chem..

[54] Rasool S., Raza M.A., Manzoor F., Kanwal Z., Riaz S., Iqbal M.J., Naseem S. (2020). Biosynthesis, characterization and anti-dengue vector activity of silver nanoparticles prepared from *Azadirachta indica* and *Citrullus colocynthis*: Biosynthesis of silver nanoparticles. R. Soc. Open Sci..

[55] Prabhu S., Poulose E.K. (2012). Silver nanoparticles: Mechanism of antimicrobial action, synthesis, medical applications, and toxicity effects. Int. Nano Lett..

[56] Klueh U., Wagner V., Kelly S., Johnson A., Bryers J.D. (2000). Efficacy of silver-coated fabric to prevent bacterial colonization and subsequent device-based biofilm formation. J. Biomed. Mater. Res. Off. J. Soc. Biomater. Jpn. Soc. Biomater. Aust. Soc. Biomater. Korean Soc. Biomater..

[57] Dibrov P., Dzioba J., Gosink K.K., Haäse C.C. (2002). Chemiosmotic mechanism of antimicrobial activity of Ag^+^ in *Vibrio cholerae*. Antimicrob. Agents Chemother..

[58] Niño-Martínez N., Salas Orozco M.F., Martínez-Castañón G.-A., Torres Méndez F., Ruiz F. (2019). Molecular mechanisms of bacterial resistance to metal and metal oxide nanoparticles. Int. J. Mol. Sci..

